# Supplementary material for: Red Ginseng Attenuates the Hepatic Cellular Senescence in Aged Mice
Source: Biology (Basel). 2024 Jan 8;13(1):36. doi: 10.3390/biology13010036 (PMC10813250; doi:10.3390/biology13010036)
Supplement: Supplementary file 1 [file biology-13-00036-s001.zip › biology-2755570-supplementary.pdf]

Supplementary material

Figures S1–S4: details of western blot membranes

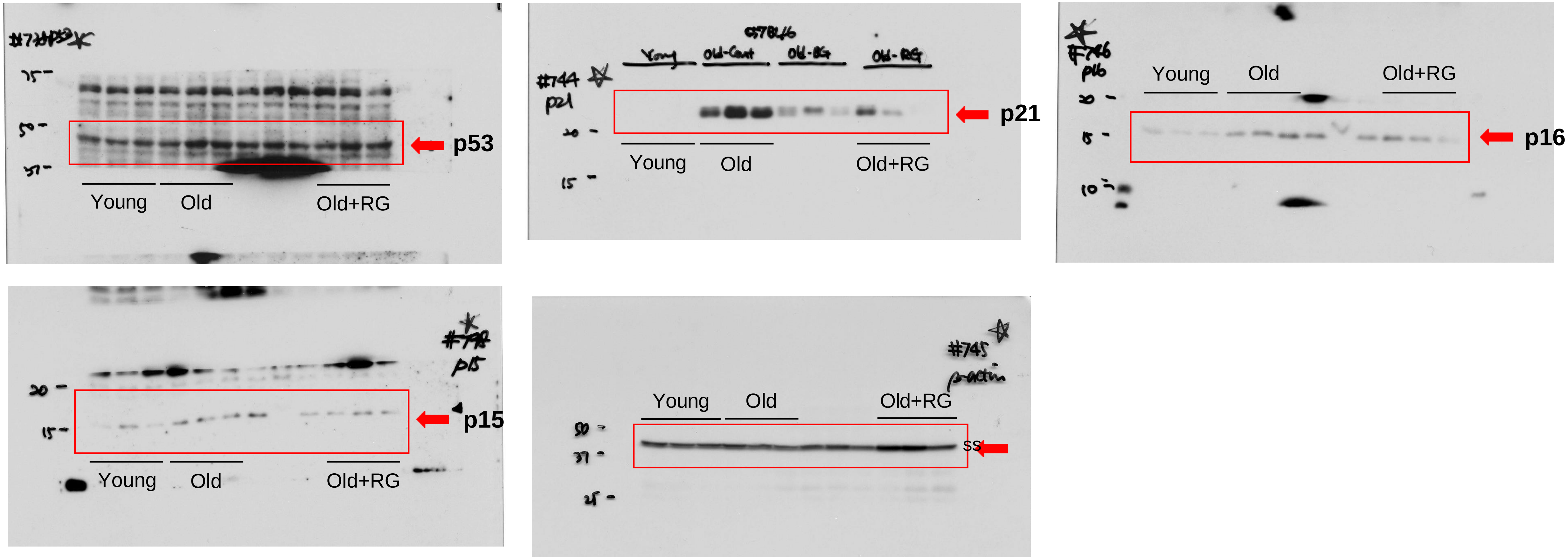

Supplemental Figure S1. Total liver tissue lysates were immunoblotted with cellular senescence-related markers: p53, p21, p16, and p15. (Supports Figure 2A).

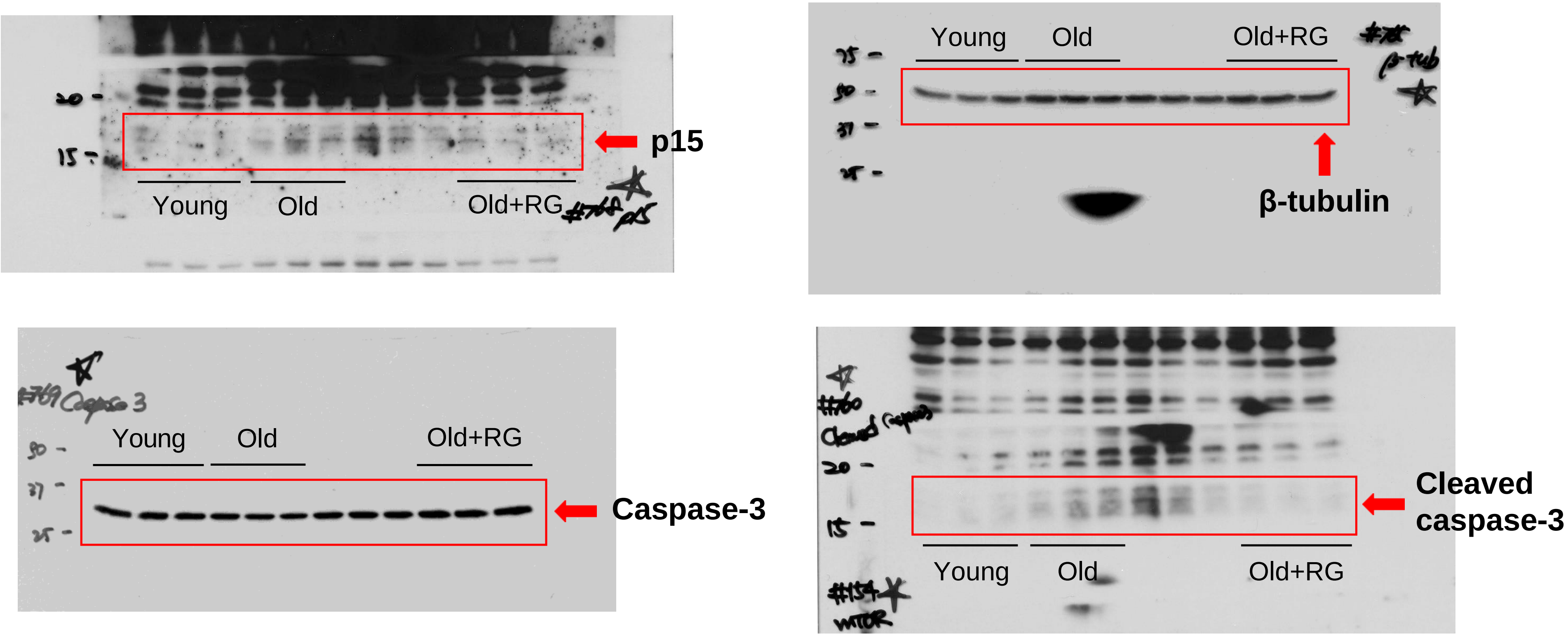

Supplemental Figure S2. Primary hepatocytes were isolated from Young, Old, and Old + RG mice, followed by immunoblotting with p15 and cleaved caspase-3 and caspase-3.(Supports Figure 2B, 2C).

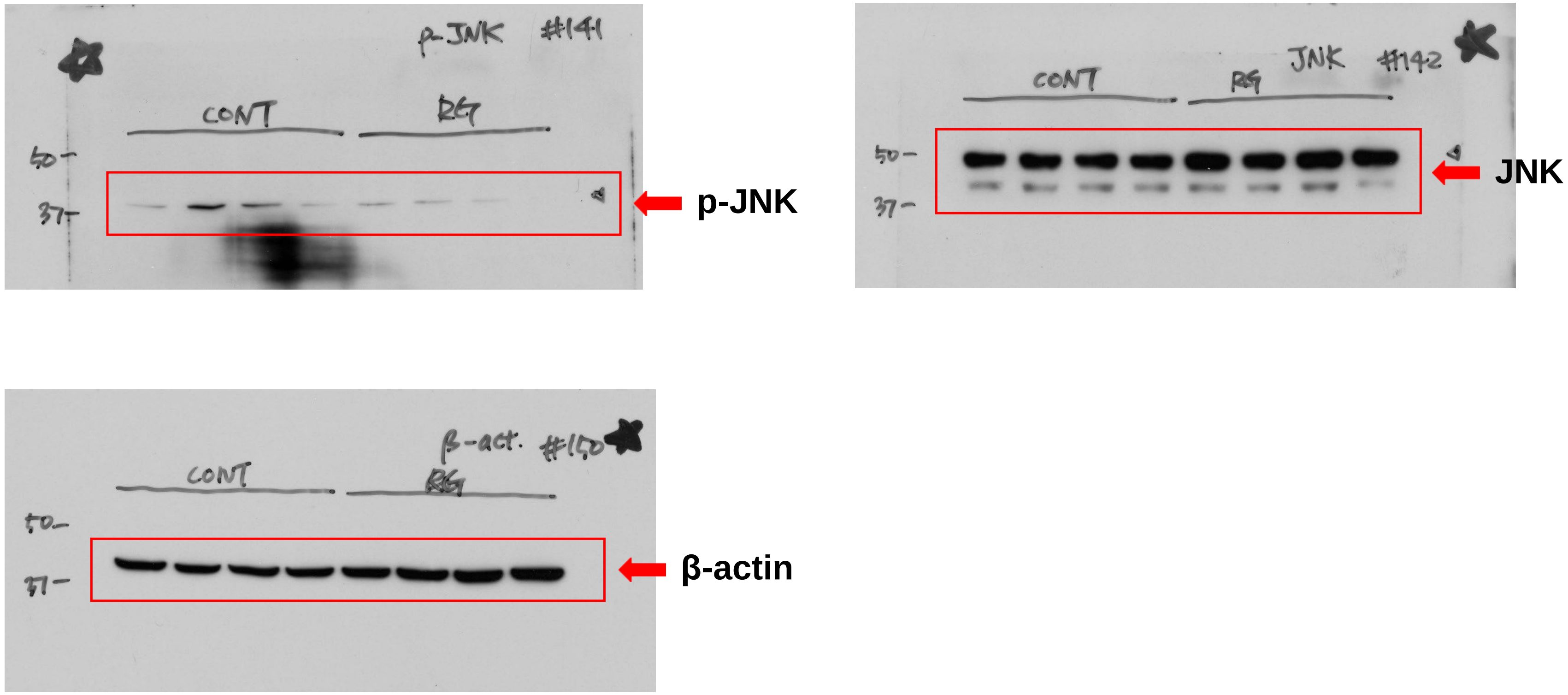

Supplemental Figure S3. The immunoblots were quantified by normalizing p-JNK and JNK to β-actin and p-JNK to total JNK. (Supports Figure 4A).

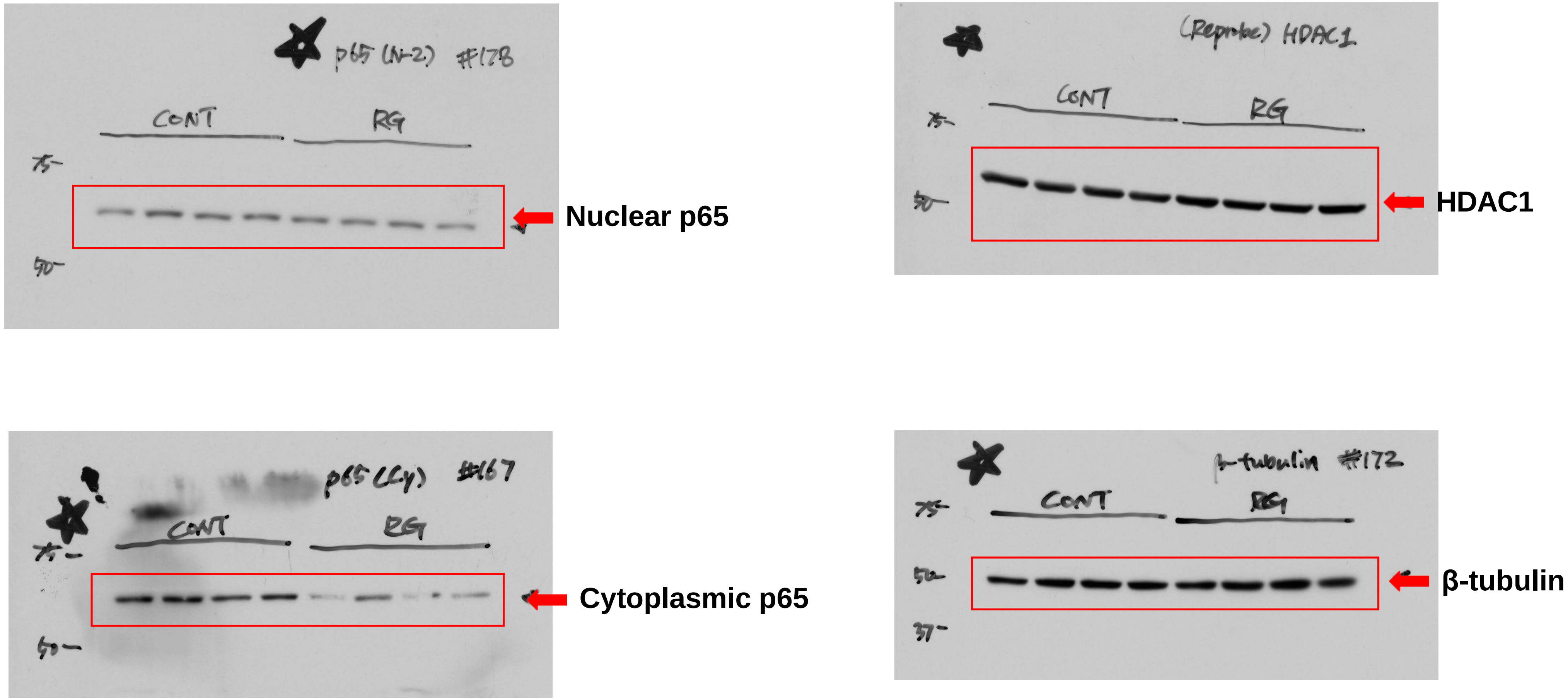

Supplemental Figure S4. The nuclear and cytoplasmic fractions were fractionated from the mouse liver tissues. The quanti-fication of immunoblots was performed by the normalization of nuclear p65 to HDAC1, and cy-toplasmic p65 to β-tubulin. (Supports Figure 4B).
